# Notch system is differentially expressed and activated in pituitary adenomas of distinct histotype, tumor cell lines and normal pituitaries

**DOI:** 10.18632/oncotarget.19046

**Published:** 2017-07-06

**Authors:** Sofia Perrone, Lautaro Zubeldia-Brenner, Elias Gazza, Gianina Demarchi, Leticia Baccarini, Agustin Baricalla, Freya Mertens, Guillermina Luque, Hugo Vankelecom, Silvia Berner, Damasia Becu-Villalobos, Carolina Cristina

**Affiliations:** ^1^ Centro de Investigaciones y Transferencia del Noroeste de la Provincia de Buenos Aires, CITNOBA (UNNOBA-CONICET), Universidad Nacional del Noroeste de la Provincia de Buenos Aires, Pergamino, 2700 Buenos Aires, Argentina; ^2^ Instituto de Biología y Medicina Experimental, IBYME-CONICET, 1428 Buenos Aires, Argentina; ^3^ Department of Development and Regeneration, Cluster Stem Cell Biology and Embryology, Research Unit of Stem Cell Research, KU Leuven (University of Leuven), Campus Gasthuisberg O&N4, B-3000 Leuven, Belgium; ^4^ Servicio de Neurocirugía, Clínica Santa Isabel, C1406GZJ Buenos Aires, Argentina

**Keywords:** Notch, pituitary, corticotropinoma, prolactinoma, Jagged1

## Abstract

Pituitary adenomas are among the most frequent intracranial neoplasms and treatment depends on tumor subtype and clinical features. Unfortunately, non responder cases occur, then new molecular targets are needed.

Notch system component expression and activation data are scarce in pituitary tumorigenesis, we therefore aimed to characterize Notch system in pituitary tumors of different histotype. In human pituitary adenomas we showed *NOTCH1-4* receptors, *JAGGED1* ligand and *HES1* target gene expression with positive correlations between *NOTCH1,2,4* and *HES1*, and *NOTCH3* and *JAGGED1* denoting Notch system activation in a subset of tumors. Importantly, NOTCH3 positive cells were higher in corticotropinomas and somatotropinomas compared to non functioning adenomas. In accordance, Notch activation was evidenced in AtT20 tumor corticotropes, with higher levels of NOTCH1-3 active domains, *Jagged1* and *Hes1* compared to normal pituitary.

In the prolactinoma cell lines GH3 and MMQ, *in vivo* GH3 tumors and normal glands, Notch system activation was lower than in corticotropes. In MMQ cells only the NOTCH2 active domain was increased, whereas NOTCH1 active domain was higher in GH3 tumors. High levels of *Jagged1* and *Dll1* were found solely in GH3 cells, and *Hes1*, *Hey1* and *Hey2* were expressed in a model dependent pattern.

Prolactinomas harbored by lacDrd2KO mice expressed high levels of NOTCH1 active domain and reduced *Hes1*.

We show a differential expression of Notch system components in tumoral and normal pituitaries and specific Notch system involvement depending on adenoma histotype, with higher activation in corticotropinomas. These data suggest that targeting Notch pathway may benefit non responder pituitary adenomas.

## INTRODUCTION

Pituitary adenomas are among the most frequent intracranial neoplasms, with a prevalence estimated at 22.5% by autopsy and magnetic resonance imaging studies [1, 2]. They are classified as functional or non functioning tumors, the former with clinical specific symptoms due to increased hormonal secretion and activity. Non functioning pituitary adenomas, with no symptoms or signs secondary to hormone over-secretion, are identified instead by mass effect or incidentally found in autopsy [1, 3, 4]. Treatment depends on tumor subtype, size and mass compression on cerebral neighboring tissue. Surgery is the first line of treatment for non functioning adenomas. Instead, effective medical therapy has been demonstrated in functioning adenomas, in particular in prolactin and growth hormone secreting pituitary tumors [5]. Unfortunately, non responder cases occur in all tumor subtypes [6]. Therefore, new molecular targets are needed to improve treatments.

The Notch system is conserved across species [7, 8]. It has been extensively characterized as a regulator of cell fate decisions in a variety of organisms and tissues, and participates in tumor generation so that its presence and involvement in pituitary tumor development merits a comprehensive study.

There are four Notch receptors (Notch1–4) in mammals. They are single pass type I transmembrane molecules coded by a single precursor that is activated by post-transductional proteolytic cleavage resulting in a non-covalently linked heterodimer composed of an extracellular fragment and a transmembrane-intracellular subunit [9].

There are five mammalian Notch ligands, three of which belong to the Delta family (Dll1, Dll3 and Dll4) and two to the Jagged family (Jagged1 and Jagged2). Notch ligands are also transmembrane proteins so that Notch pathway activation involves cell to cell contact. Receptor–ligand interaction triggers crucial signaling cleavages within the Notch receptor [10]. The clipping of the extracellular domain creates a membrane–tethered intermediate which is a substrate for an intramembrane cleaving protease, the γ-secretase [11]. γ-secretase-induced cleavage finally releases the Notch intracellular domain (NICD) from the membrane, and the released NICD translocates directly to the nucleus, where it forms a transcriptional complex with the DNA binding protein CSL, Mastermind and transcriptional coactivators to drive the expression of Notch target genes [7, 8]. Target genes regulated by Notch are highly dependent on cell type and can include genes whose products are involved in fundamental aspects of cell biology, such as cell cycle regulation [12, 13], cellular differentiation, and metabolism [14]. Common targets of the pathway include the Hes and Hey families of transcription repressors [15–17] as well as Myc transcription factor [14, 18, 19]. The binding and function of Notch on DNA involves a rapid and dynamic process controlled by phosphorylation and ubiquitination and subsequent proteasomal degradation [20, 21], which shuts off the pathway. In the absence of NICD, CSL forms complexes with a variety of corepressors to suppress the transcription of Notch target genes [7, 8].

Given the important and widespread roles of Notch signaling across a range of tissues, it is not surprising that abnormal Notch signaling is involved in several inherited human diseases [22, 23]. Furthermore, Notch malfunction has been associated with diseases linked to changes in cell fate and cell proliferation including cancer [22]. These syndromes present a broad range of clinical symptoms, emphasizing the highly pleiotropic nature of Notch. Moreover, both solid tumors and leukemias have been associated with deregulation of the Notch system in which, depending on the context, it can act both as an oncogene or a tumor suppressor. Notch signaling is overexpressed or constitutively activated in colorectal cancer partly because of mutations in regulators of Notch signaling [24–26]. In particular, Jagged1 expressed in tumor cells or produced by endothelial cells, is thought to be a key ligand for Notch activation in colorectal cancer cells [27, 28]. Notch signaling plays a crucial role in the early stages of colorectal cancer development by controlling the fate of stem cells and cancer stem cells [29]. In mammals, adult stem cell subpopulation sustains tissue homeostasis granting cell replacement during aging or damage [30]. Similarly, different cell populations were described in tumors in which has been identified a small fraction of tumor-initiating cells, representing the so-called cancer stem cells [31]. Notch system also participates at the later stages of tumor invasion and metastasis [29]. A number of studies have confirmed that activation of Notch signaling also plays an oncogenic role in breast cancer [32, 33]. In contrast, more recent studies indicate that hyperactivation of Notch3 may be detrimental to breast cancer cells by inducing senescence [34].

Various components of the Notch pathway are expressed during pituitary development, including Notch2 and Notch3 receptors, the ligand Jagged 1 and the downstream effector Hes1 [35–37]. Interestingly, in the anterior pituitary of adult mice, Notch receptor genes were found to be expressed in side population cells (SP), a cellular fraction which is enriched in stem/ progenitor cells [38–40]. In the adult rat pituitary, Tando and colleagues determined that the Notch system is involved in follicle stellate- S-100 positive cell proliferation, and that *Notch1*, *Notch2*, and *Jagged1* colocalized with S-100 protein, while *Jagged2* expression was described in melanotropes [41]. Recently, the same group also described that Notch2 activation in the pituitary gland needed E-cadherin mediated cell attachment [42].

In pituitary tumorigenesis, published data related to Notch system expression and functions are scarce. GeneChip microarrays and proteomics analyses demonstrated increased expression of *NOTCH3* in non functioning and prolactin secreting adenomas in humans while in somatotropinomas a significantly reduced expression of *NOTCH3* was found [43, 44]. Furthermore, microarray analysis performed in the fractioned SP and main population from human GH and non functioning pituitary adenomas cells showed more than 1.5 fold increased expression of components of the Notch system in the SP, including *HES1*, *JAG1* and *NOTCH* paralogs [40]. It is known that *Notch* and *Wnt* pathway genes as other key markers, represent not only stem cell signaling systems but also regulatory circuits known for their critical role in pituitary embryonic development [45].

Notch3 and Jagged1 were also overexpressed in human clinically non functioning pituitary adenomas compared to normal pituitary gland [46, 47], while no significant differences were determined for prolactin or growth hormone secreting adenomas in that study [46].

Evidence points to an association of increased activation of the Notch system with more aggressive pituitary adenomas. However, there is no complete description of all Notch receptors, ligands and downstream effectors in the normal and pathological pituitary gland. Indeed, there are only few studies evaluating Notch system in human ACTH secreting adenomas [48] or prolactinoma models. There is definitely a potential therapeutic benefit for targeting Notch in tumorigenesis, as evidence in pituitary adenomas is lacking. But, because Notch function and system components can substantially differ and be dependent on cell type and tissue, and specific for each type of cancer, it is vital to characterize gene expression and activation in each adenoma type.

Therefore, in the present study we decided to evaluate the expression of the different Notch receptors and other components of the system in a comparative manner in tumoral and normal pituitaries in human and rodent samples. In this way, we aimed to elucidate Notch system significance in pituitary tumor development in search of new targets for the treatment of adenomas with resistance or intolerance to pharmacological therapy in which no alternatives exist other than pituitary surgery.

## RESULTS

### Notch signaling component expression in human pituitary adenomas

**Figure 1:**
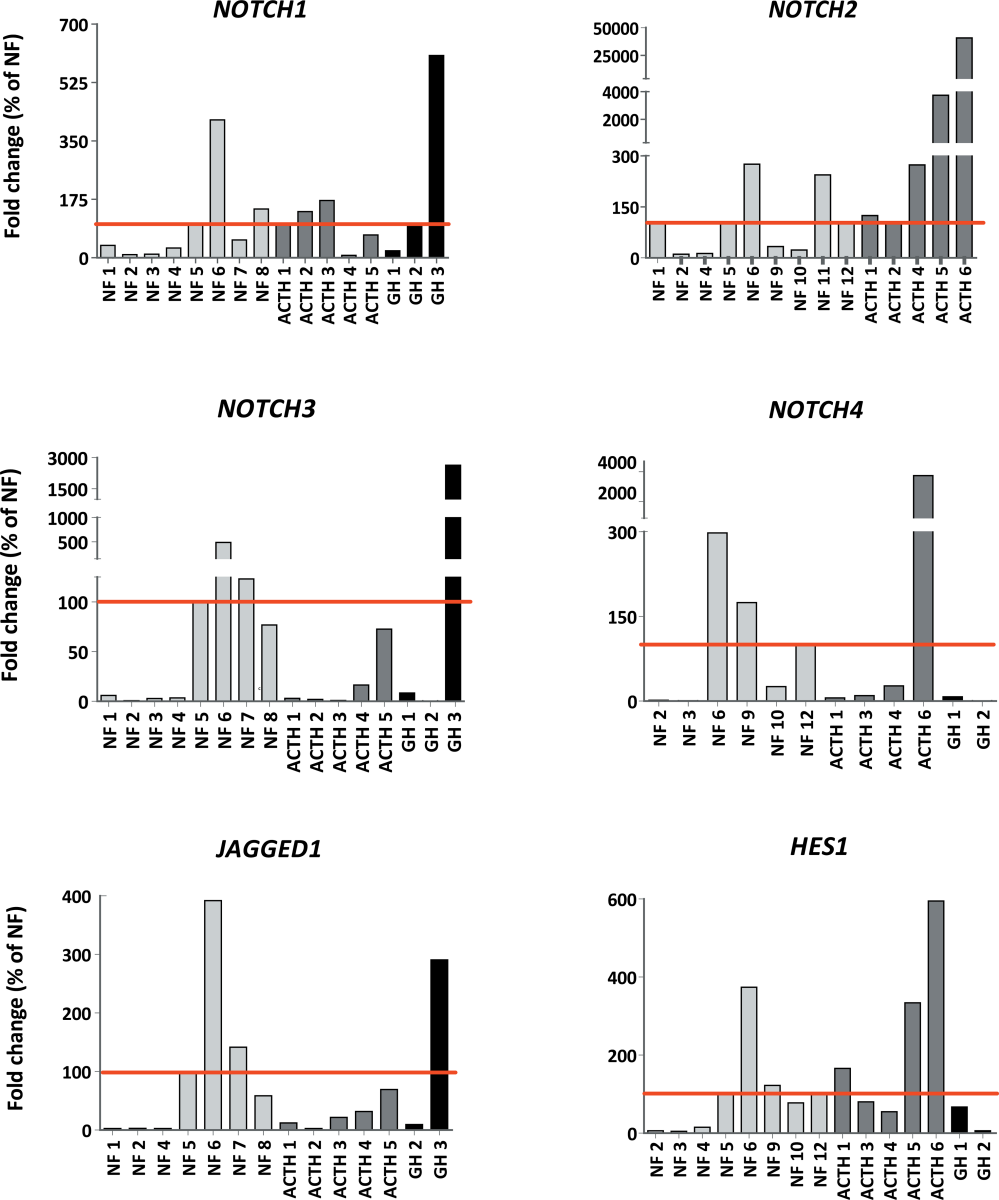
Notch system components are expressed in human pituitary adenomas mRNA expression of *NOTCH1-4* receptors, *JAGGED1* ligand and *HES1* target gene were determined by qRT-PCR. Gene levels normalized to the housekeeping gene *Gadph* are shown as percentage of change of NF average (which was considered 100%) (NF = non functioning adenoma; ACTH = corticotropinoma; GH = somatotropinoma).

In human pituitary adenomas *NOTCH1-4* mRNA expression was detected in all tumors analyzed (Table 1), with variable levels among the same adenoma type (Figure 1A–1D). The expression of *JAGGED1* and *HES1* was also determined in samples in which sufficient RNA was available and variable levels of expression were quantified independently of tumor type (Figure 1E–1F).

**Table 1:** Clinical and tumor features from human samples used in qRT-PCR experiments

Histotype	Age	Sex	Micro / macroadenoma	Recurrence	Ki67
NF1	47	M	macroadenoma	no	3, 0
NF2	62	F	macroadenoma	no	> 3
NF3	42	F	macroadenoma	no	4, 5
NF4	45	M	macroadenoma	no	< 1
NF5		M	-	no	5
NF6	67	M	macroadenoma	-	-
NF7		M	-	no	-
NF8	41	F	macroadenoma	no	1, 8
NF9	42	F	macroadenoma	no	4, 5
NF10	46	F	-	no	< 1
NF11	68	F	macroadenoma	yes	4
NF12	58	F	macroadenoma	no	3
ACTH1	28	F	microadenoma	no	< 1
ACTH2	56	M	macroadenoma	no	3, 4
ACTH3	25	F	microadenoma	no	< 1
ACTH4	67	F	macroadenoma	yes	9
ACTH5	38	M	macroadenoma	no	1, 5
ACTH6	65	F	macroadenoma	yes	11, 2
GH1	36	F	-	-	-
GH2	60	F	macroadenoma	no	1
GH3	35	M	macroadenoma	yes	8, 0

Age, sex, recurrence and tumor size, histotype and Ki67 proliferation index from human samples used in qRT-PCR experiments. *N* = 21.

**Figure 2:**
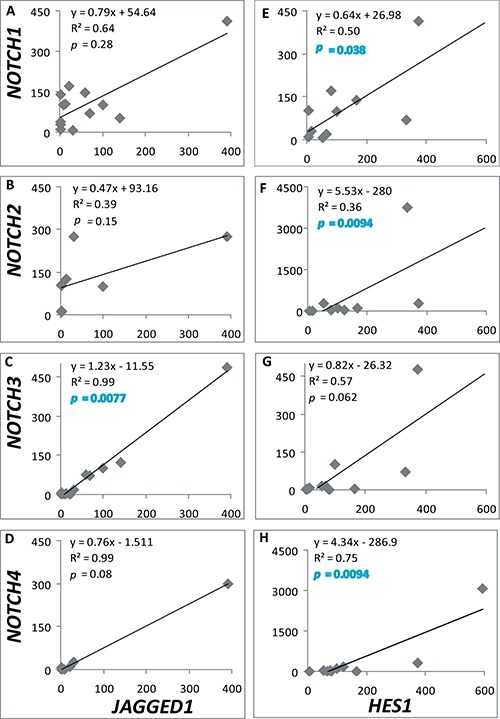
*NOTCH-JAGGED1* and *NOTCH-HES1* correlations Relation between mRNA levels of *NOTCH1-4* and *JAGGED1* (*n = 13, 8, 13, 6* (**A**–**D**)) or *HES1* (*n* = 11, 10, 11, 12 (**E**–**H**)) was determined in all adenomas tested. The equation of linear regression, R2 coefficient of determination and coefficient of Spearman are shown in each graph. *p* ≤ 0.05 denotes a significant correlation.

Remarkably, positive correlations between the expression of *NOTCH1-2,4* and the *HES1* target gene, and between *NOTCH3* with the ligand *JAGGED1* were found in the cohort of samples used when all adenomas were considered, independently of tumor histotype (Figure 2). These significant correlations clearly denote activation of the Notch system in a subset of pituitary adenomas.

Instead, no correlation between Ki-67 and any of the Notch system components analyzed was found (data not shown). Correlations of gene expression and tumor recurrence or extrasellar extension could not be performed as only 4 were recurrent tumors, and most were macroadenomas (Table 1).

**Figure 3:**
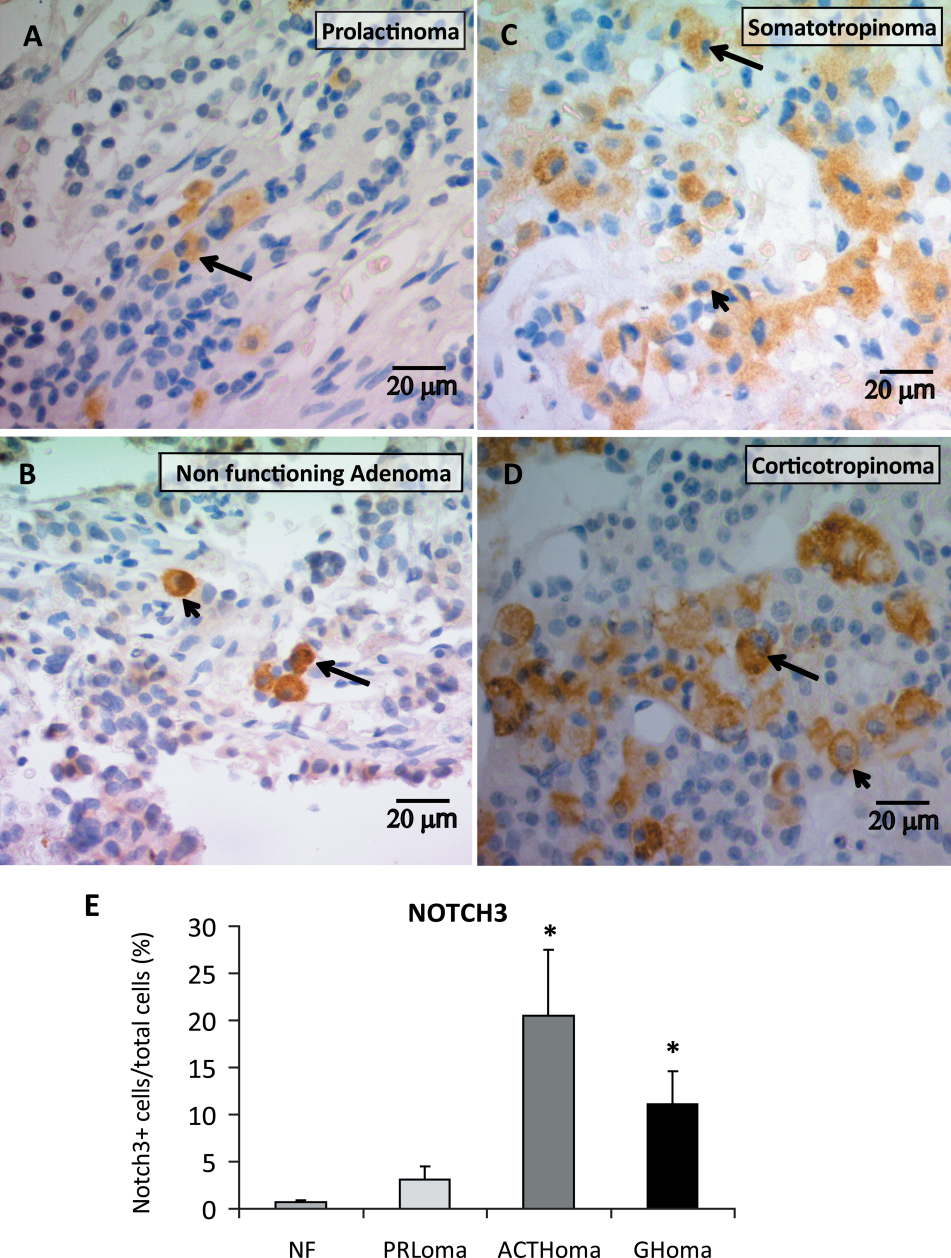
NOTCH3 protein expression determined by immunohistochemistry Percentage of positive cells, distribution and cellular localization in human pituitary adenomas. Representative images of NOTCH3 stained cells, (**A**–**B**) NOTCH3+ cells were isolated within prolactinomas and NF pituitary tumors, and in the GH and ACTH tumors (**C**–**D**) specific stained groups of positive cells were evidenced. NOTCH staining was visualized in membranes and cytoplasms of human adenoma cells. Arrows indicate staining in cytoplasm, and arrowheads in cell membranes. (**E**) Average of the percentage of NOTCH3+ cells in relation to total cells for Non Functioning adenomas (NF), prolactinomas (PRLoma), corticotropinomas (ACTHoma) and somatotropinomas (GHoma). *N* = 3,2,3,4 patients, respectively. *P ≤ 0.05 vs. NF.

To evaluate Notch protein expression, distribution and cellular localization in human pituitary adenomas (see Table 2) we performed NOTCH3 immunohistochemistry and found that non functioning adenomas, prolactinomas, somatotropinomas and corticotropinomas expressed NOTCH3 protein in the cytoplasm and membrane of tumor cells (Figure 3A–3D). In prolactinomas and non functioning adenomas NOTCH3 cells were scattered and isolated (Figure 3A, 3B). Conversely, in somatotropinomas and corticotropinomas, NOTCH3-positive cells were observed organized in clusters with particularly intense staining in ACTH tumors (Figure 3C, 3D). Quantitation of percentage of NOTCH3 positive cells per total cells showed higher levels in corticotropinomas and somatotropinomas compared to non functioning adenomas (Figure 3E).

**Table 2:** Clinical and tumor features from human samples used in immunohistochemistry experiment

Histotype	Age	Sex	Micro / macroadenoma	Recurrence	Ki67
ACTH-a		F	microadenoma	no	−
ACTH-b	46	F	microadenoma	no	< 1
ACTH-c		F	microadenoma	no	−
GH-a	49	F	macroadenoma	no	3
GH-b	58	F	microadenoma	no	< 1
GH-c	47	F	–	no	3.0
GH-d	26	M	macroadenoma	yes	3, 5
PRL-a	50	F	macroadenoma	no	12
PRL-b	50	M	macroadenoma	no	–
NF-a	37	F	microadenoma	no	–
NF-b		F	macroadenoma	no	–
NF-c	67	M	macroadenoma	no	–

Age, sex, recurrence and tumor size, histotype and Ki67 proliferation index from human samples used in NOTCH3 immunohistochemistry experiment. *N* = 12.

### Notch system is active in the corticotropinoma cell line AtT20

**Figure 4:**
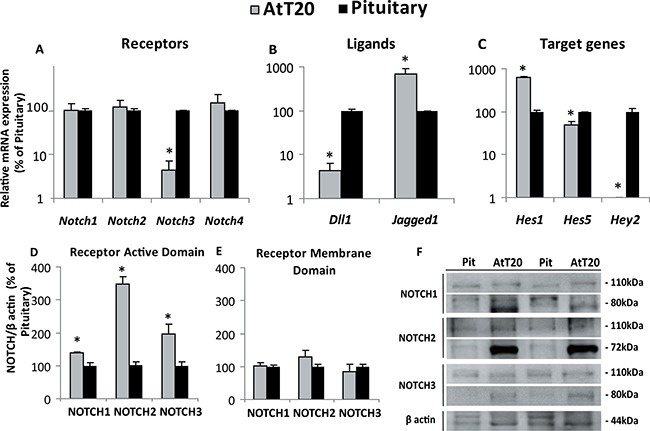
Notch system component expression in mouse corticotrope tumor cells and normal pituitaries (**A**–**C**) Gene expression normalized to *Gapdh* in AtT20 cells and mouse pituitaries determined by qRT-PCR and expressed as percentage of change of the normal pituitaries which were considered 100%. *p = 0.0003 for *Notch3, Notch1,2,4* NS, *n* = 3–5; 3–8 for AtT20 and pituitary respectively (A), *p* = 0.0111 and *p* = 0.0105 for the Notch ligands *Dll1 and Jagged1*, respectively vs pituitary and (B) *p* = 0.0016, 0.0184 and 0.0032 for the Notch target genes *Hes1, Hes5 and Hey2*, respectively, vs. pituitary *n* = 2–3; 3 for AtT20 and pituitary respectively (C). (**D**, **E**) Active and membrane domains of NOTCH1-3 receptor levels determined by Western Blot: Bars show the mean of the receptor normalized to β actin levels, expressed as the percentage of normal mouse pituitary. *p = 0.026 for NOTCH1, *p <* 0.0001 for NOTCH2 and *p* = 0.0146 for NOTCH3 vs. pituitary, for the active domains (80KDa) *n* = 3–6; 5–7 for AtT20 and pituitary respectively (D). No significant differences were found for the membrane domain (110 KDa) *n* = 3–6; 3–7 for AtT20 and pituitary respectively (E). Representative Western blots showing the active and membrane domains of NOTCH receptors in normal pituitaries (Pit) and ATt20 cells (**F**).

mRNA expression of the components of the Notch system was determined in the AtT20 tumoral corticotrope cell line by qRT-PCR and compared with normal mouse pituitaries. *Notch1, Notch2 and Notch4* mRNA expression levels were similar in AtT20 cells and normal adult pituitaries whereas *Notch3* mRNA expression was reduced in corticotrope tumor cells in relation to normal mouse pituitary cells (Figure 4A).

The Notch ligands *Dll1* and *Jagged1* were also analyzed and significantly increased *Jagged1* and decreased *Dll1* mRNA levels in AtT20 cells compared to normal pituitary cells were found (Figure 4B). On the other hand, Notch target genes *Hes1, Hes5* and *Hey2* were expressed with a particular pattern for each gene, with higher *Hes1* expression in tumor corticotropes and reduced *Hes5* and *Hey2* mRNA levels compared with normal pituitary glands, *Hey2* being almost undetectable in ATt20 cells. These results could be related with the specific function they might have in normal and tumoral cells (Figure 4C).

At the protein level, expression of the active NOTCH intracellular domains of NOTCH1-3 receptors were significantly increased in the tumoral cells suggesting these receptors are activated and involved in the tumorigenic process of the corticotropinoma cell line (Figure 4D, 4F). Instead, the membrane domain of the Notch receptor showed no differences between the tumor cell line and the normal pituitary (Figure 4E, 4F).

### The Notch system is expressed in GH3 and MMQ cell lines and in experimental prolactinomas

In order to determine the relative expression of each Notch receptor in different tumoral lactotrope cell lines, we first performed qRT-PCR of the four *Notch* receptors in the rat prolactinoma MMQ cells (prolactin secreting cells) and GH3 (growth hormone and prolactin secreting cells). We also analyzed the *in vivo* model of tumor xenografts generated by subcutaneous injection of GH3 cells in Nude mice, and compared gene expression to that of normal rat pituitary glands.

**Figure 5:**
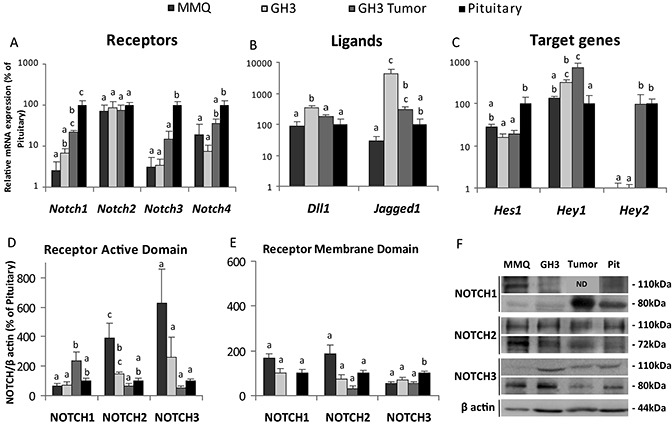
Expression pattern of Notch pathway genes and proteins in prolactinoma cell lines, tumor xenografts and normal pituitaries (**A**–**C**) Differential gene expression normalized to *Gapdh* and expressed as percentage of change of the normal pituitaries considered 100% determined by qRT-PCR. For the figures in this panel, different letters indicate significant differences (*p* ≤ 0.05) between groups. (A) *Notch1-4* receptors *n* = 4–5, 5, 4, 4–6 for MMQ, GH3, GH3 *in vivo* tumors and rat pituitaries. (B) Notch ligands *Dll1 and Jagged1 n* = 3, 3, 3, 2 and (C) Notch target genes *Hes1, Hey1 and Hey2* *n* = 3, 3, 3, 2 for MMQ, GH3, GH3 *in vivo* tumors and rat pituitaries, respectively. (**D**–**E**) Active and membrane domains of NOTCH1-3 receptors were determined by Western blot. Bars show the mean of the relation of the receptor levels to β actin expression, showed as the percentage of normal rat pituitary. (D) The active domains (80KDa): *n* = 5–6; 5, 3, 6. (E) The membrane domains (110KDa): *n* = 3–6, 2–5, 0–3, 3–6 for MMQ, GH3, GH3 tumors and normal rat pituitaries. (**F**) Representative Western blots showing the active and membrane domains of NOTCH receptors in MMQ, GH3, GH3 *in vivo* tumors and normal pituitaries.

GH3 and MMQ cells, and also GH3 *in vivo* tumors expressed *Notch* RNA messengers, and with the exception of *Notch2* in which no differences with normal pituitaries were found, the rest of *Notch* paralogs showed lower levels of mRNA in tumoral cell lines. GH3 xenografts instead, showed only reduced *Notch3* levels compared to control pituitaries (Figure 5A).

Nevertheless, we found that the intracellular active domain of NOTCH2 was significantly increased in the MMQ prolactinoma cell line. Instead, NOTCH1 active domain expression was higher in GH3 *in vivo* tumors compared to prolactin secreting cell lines while NOTCH3 active domain showed no differences (Figure 5D, 5F). The membrane domains of the NOTCH receptors showed no differences, with the exception of NOTCH3 which was lower in prolactin secreting cell lines and tumor xenografts compared to normal pituitaries (Figure 5E, 5F), in correlation with its mRNA expression.

The expression of the ligands *Jagged1* and *Dll1* was higher in GH3 cells than in control cells, (Figure 5B). Therefore, in the light of the results presented herein showing high levels of active Notch receptors and the Notch ligands *Jagged1* and *Dll1* in GH3 cells or *Jagged1* in AtT20 cells, an activation of the Notch system in tumoral pituitary cell lines can be inferred.

Additionally, analysis of the expression of the target genes *Hes1, Hey1 and Hey2* suggested variable activation which differed substantially between clonal cell lines, tumor xenografts and normal pituitaries. This variability may reflect different effectors and actions of the Notch system in normal and tumor cells. In particular, *Hes1* expression predominated in normal rat pituitary when compared to prolactin secreting cell lines and xenografts, *Hey1* showed higher levels in the GH3 tumor relative to MMQ cells and normal gland, and the target *Hey2* was detected in the GH3 xenograft model and in the normal pituitary but was almost absent in the tumor MMQ and GH3 cell lines, similarly to results found in AtT20 cells for this transcription factor (Figure 5C).

Differences in GH3 *in vivo* tumors and GH3 cells were also noteworthy. Higher levels of NICD1 and lower levels of NICD2 were found in GH3 xenografts compared to the cell line, as well as reduced expression of *Dll1* and an increment of *Hey2*.

Comparing results between cell lines established from adenomas of different histotypes, it became evident that the Notch system was more activated in ATt20 compared to GH3 and MMQ cells. First, the active intracellular domains of Notch1-3 were increased in this corticotrope cell line, while no increase in the activated paralogs was found in GH3 cells, and only NICD2 but not NICD1 or NICD3 was increased in MMQ cells. Secondly, the canonical target *Hes1* was increased in ATt20, but decreased in GH3 cells. And finally, Notch mRNA expression (*Notch1, 3 and 4*) was consistently decreased in GH3 and MMQ cells while only *Notch3* was decreased in ATt20. Consistent among the three cell lines a dramatic decrease in *Hey2* mRNA in comparison to normal pituitaries was determined.

### Notch system is upregulated in prolactinomas harbored by lacDrd2KO mice *in vivo*

**Figure 6:**
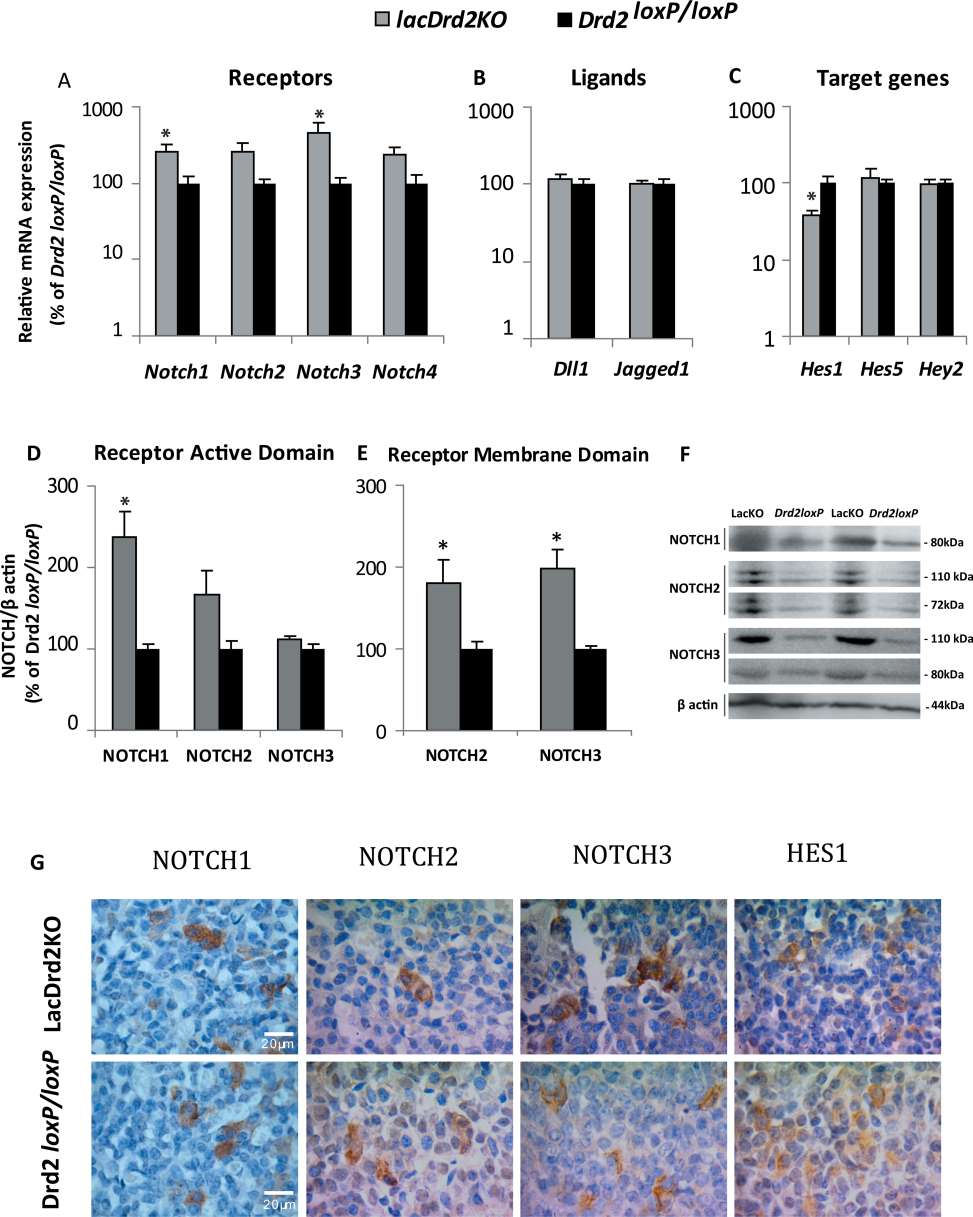
Prolactinomas of lacDrd2KO female mice have an active Notch system (**A**–**C**) Gene expression determined by qRT-PCR in pituitaries of lacDrd2KO and *Drd2l^oxP/loxP^* counterparts normalized to *Gapdh* and expressed as percentage of change of the *Drd2^loxP/loxP^* pituitaries considered 100%. Bars show mean values. *Notch1* *p = 0.0431, *Notch3* *p = 0.0089 vs. *Drd2^loxp/loxp^*; *Notch2, 4* NS, *n* = 7, 5 for lacDrd2KO and *Drd2^loxP/loxP^* mice, respectively (A). Notch ligands *Dll1* and *Jagged1* NS (B), and Notch target genes *Hes1* (*p = 0.0106 lacDrd2KO vs. *Drd2^loxP/loxP^*)*, Hes5* and *Hey2*, NS (C); *n* = 7, 5 for *lacDrd2KO* and *Drd2^loxP/loxP^* mice, respectively. (**D**–**E**) The expression of the active and membrane domains of NOTCH1-3 receptors was determined by Western blot, related to the correspondent β actin levels, and expressed as the percentage of *Drd2^loxP/loxP^* pituitary average. *p = 0.01 for NOTCH1, NOTCH2, 3 NS active domain, *p* =0.0604 and *p* = 0.0042 for NOTCH2 and NOTCH3 membrane domains, respectively for lacDrd2KO vs *Drd2^loxP/loxP^ m*ice. *n* = 4, 3–4 for lacDrd2KO and *Drd2^loxP/loxP^*. Representative Western blots showing the active and membrane domains of NOTCH receptors in lacDrd2KO and *Drd2^loxP/loxP^* (**F**). Representative microphotographies of NOTCH1-3 and HES1 Immunohistochemistry performed in lacDrd2KO and *Drd2^loxP/loxP^* mice (**G**).

Clear upregulation of the Notch system was observed in prolactinoma-bearing pituitaries of female mice lacking the dopamine D2 receptor exclusively in lactotropes, compared to their counterparts, pituitaries from *Drd2^loxP/loxP^* mice. *Notch1* and *Notch3* receptor mRNA levels were significantly increased in the lacDrd2KO pituitaries (Figure 6A). Moreover, significantly increased NOTCH1 active domain and NOTCH2 and NOTCH3 membrane domains were found in the tumoral pituitaries by Western blot assessment (Figure 6D–6F).

On the other hand, and opposite to the evidence we found in pituitary tumor cell lines, no differences were detected in the expression of pituitary *Dll1* or *Jagged1* Notch ligands between lacDrd2KO and control mice (Figure 6B). Instead, *Hes1* target gene was significantly reduced in the pituitaries of lacDrd2KO in comparison with *Drd2^loxP/loxP^* mice while similar expression levels of the other Notch target genes measured, *Hes5* and *Hey2* were observed (Figure 6C).

Even though marked differences were found for gene and protein Notch expression levels, the cellular localization of the receptors was similar for both genotypes. NOTCH1-3 protein expression evidenced by immunohistochemistry was found in membranes and cytoplasms of both lacDrd2KO and *Drd2^loxP/loxP^* pituitary cells. Furthermore, no differences between genotypes were observed in the staining pattern of pituitary HES1, which was localized in the cytoplasm (Figure 6G).

## DISCUSSION

The Notch system plays a fundamental role in normal development and cell-fate determination in a variety of multicellular organisms. It modulates specific cell cycle inhibitor expression promoting cell cycle exit and balancing proliferation and differentiation mainly acting through *Hes1* [36]. In pituitary development it regulates cell precursor number, organ size, cell differentiation and fate [49].

Genes that are important during development or differentiation often contribute to tumor promotion and survival when they become deregulated. In this context, Notch signaling has been associated with several human cancers, including acute T cell lymphoblastic leukemia, cervical, breast and prostate cancers, and lung and hepatocellular carcinoma [50–52]. Interestingly, Notch activation can be growth-promoting or inhibitory, depending on the cellular context. For example, in non-small cell lung cancer, Notch function seems to be oncogenic, and γ-secretase inhibitors, which inhibit Notch receptor activation, show antitumor activity both *in vitro* and *in vivo* [53]. In human colon cancer Notch1-related signaling also positively regulates tumor growth by promoting proliferation and survival of cancer stem cells and colon cancer cells [54]. Conversely, in prostate cancer Notch can be considered a tumor suppressor particularly at the onset of tumorigenesis, when the Notch target gene *Hey1* is activated. Hey1 as a co-factor of androgen receptor can inhibit androgen dependent targets, including those involved in prostate cancer progression [55]. Moreover, different Notch paralogs may have different actions in cancerous transformation; for example, it has been described that NOTCH1 and NOTCH3 have disparate roles from NOTCH2 in bladder cancer [56].

Notch signaling has also been implicated in the pathogenesis of human pituitary adenomas. Upregulation of *NOTCH3* gene expression was described in non functioning pituitary adenomas [43, 46, 47], and in prolactinomas [44] compared to normal pituitaries. In accordance, increased expression of the Notch ligand Jagged1 was found in non functioning pituitary adenomas compared to normal human pituitary tissue [46]. Furthermore, *NOTCH2* expression was found increased in the Hoechst high efflux population of stem cells named the side population (SP) in human pituitary adenomas [40], but reduced levels of *NOTCH2* mRNA were described in pituitary adenomas compared to craniopharyngiomas [48]. These data posit Notch2 and Notch3 as active players in pituitary tumorigenesis. But no complete characterization of the Notch system has been reported in human pituitary adenomas.

In a more comprehensive manner we addressed the expression of several components of the system in human pituitary tumors. In accordance with Notch3 involvement in pituitary tumorigenesis, we found NOTCH3 immunopositive cells in every human adenoma sample tested with higher levels in corticotropinomas and somatotropinomas, and a remarkable disposition of positive cells in clusters in these two adenoma types. Higher NOTCH3 staining detected in corticotropinomas suggest a more active Notch pathway in this adenoma subtype in accordance with results obtained in ATt20 cells compared to prolactinoma cell lines. In concordance with Notch activation in this pituitary tumor type, an overexpression of the ligand *Dll4* was found in corticotropinomas when compared to craniopharyngiomas [48].

Variable mRNA expression levels of *NOTCH1-4*, the Notch ligand *JAGGED1* and the target gene *HES1* were found in non functioning adenomas, corticotropinomas and somatotropinomas independently of tumor histotype. Interestingly, this is the first evidence of expression of not only *NOTCH3* but also other components of this cell signaling in human corticotropinomas.

A salient feature was the positive correlation found for most Notch paralogs with *HES1* expression when all adenomas were considered. Furthermore, and in accordance with other authors, a strong association between *NOTCH3* and *JAGGED1* was found in our cohort [46]. Both findings point to an activation of Notch signaling in some pituitary adenomas, in which the ligand JAGGED1 and the target HES1 would be paramount. Our results underscore the value of analyzing in each tumor sample a set of genes of the Notch system and their correlation, instead of performing single-gene analysis, in order to understand the participation of Notch in tumor generation. It remains to be elucidated if pituitary tumors with an active Notch system would benefit from a Notch targeted therapy. In that case, anti-Notch therapy could be an adjuvant to classic drug treatments like cabergoline or somatostatin analogs in low responder tumors with a demonstrated Notch activation.

On the other hand, we did not find any correlation between any component of the Notch system and the cellular proliferation index determined by Ki-67 levels. This feature could be associated to the many functions and processes regulated by Notch activation besides proliferation [8, 57].

Notch2 and Notch3 receptors are expressed during mouse pituitary embryogenesis and have been found to play key roles in cell specification during the gland development [35–37]. Furthermore, in adult mice, Vankelecom's group described high levels of transcripts of *Notch1* and *Hes1* in the SP of pituitary cells which display stem/progenitor characteristics [38] when compared to the expression in the main population. They also determined that *Notch2-4* and *Hes5* were predominantly accumulated in the SP of adult mouse pituitaries, and that Notch pathway activation in this compartment induced cell proliferation rate [39].

Our data in the mouse corticotropinoma cell line AtT20 reflects activation of the Notch pathway by the increased mRNA levels of the ligand *Jagged1* and of the intracellular domains of NOTCH1-3 receptors when compared to normal mouse pituitary glands. In the light of the need of the free intracellular domain of Notch receptors to activate the pathway, the high NICD/membrane domain ratio found in the corticotropinoma cell line indicates greater cleavage of the receptors, a process which is fundamental for the effect of Notch on tumor growth or maintenance. Furthermore, higher expression of the Notch target gene *Hes1*, but lower of *Hey2* and *Hes5* in ATt20 cells compared to mouse pituitaries suggested that HES1 may be a better candidate in Notch signaling in pituitary corticotropinomas and that neither HEY2 nor HES5 would participate in Notch system actions in the tumoral corticotrope cell line.

There are also few data describing the Notch system in animal models of prolactinomas, even though several components of the Notch pathway such as *NOTCH3, ASCL1* and *HES1* are altered in human prolactinomas [44].

Our comparative study in prolactinoma cells, *in vivo* model and normal glands did not reveal a substantial activation status of the Notch system in GH3 and MMQ cells, as observed in AtT20 corticotropes, when compared to normal glands, even though a complete Notch signaling system was determined with the expression of ligands, receptors and target genes. *Jagged1* levels were higher in GH3 somato-lactotrope cells than in MMQ and complete rat pituitaries; and *Dll1* was also higher in GH3 cells than MMQ, GH3 tumors and normal pituitaries. It could be hypothesized that Jagged1 is the main ligand by which Notch acts in GH3 and ATt20 but not in MMQ cells, and that Dll1 participates mainly in GH3 cells and not in the pure prolactin or corticotrope cell line. In GH3 cells, for example, it was described that the non canonical Notch ligand Dlk1 is expressed in some clones, in which it represses GH expression and secretion but does not affect prolactin production [58]. In the light of exclusive prolactin production in MMQ cells in contraposition to GH and prolactin secretion in GH3 cells, it could be inferred that Jagged1 and/or Dll1 may be involved in specific hormone secretion or cell type behavior.

Additionally, we show that Notch target gene *Hes1* and *Hey2* levels were decreased in prolactinoma cell lines, in line with a previous finding in human prolactinomas [44], while on the contrary, *Hey1* was overexpressed in GH3 cells. Interestingly, *Hey2* was nearly absent in MMQ and GH3 cells, but present in GH3 xenografts and control pituitaries. According to our data and the fact that cell to cell contact is an important aspect of Notch signaling cascade, *Hey*2 expression may be, in some way, associated to the presence of extracellular matrix components, vasculature and/or cell to cell contacts which are found in the complete pituitary and in the GH3 xenografted tumor. This observation is also in agreement with the very low expression of this gene found in AtT20 cell line. Moreover, the extracellular matrix probably plays a role in the expression of different components of the Notch system, as can be inferred when comparing GH3 *in vivo* tumors generated by GH3 inoculation, and GH3 cells. GH3 tumors showed higher activation of NOTCH1 and lower of NOTCH2 receptor than isolated GH3 somatolactotropic cells. Differences in *Dll1* ligand expression were also observed, pointing tumor vasculature and/or extracellular matrix components which are absent in cell lines, as important modulators of Notch signaling in somatoprolactinomas.

On the other hand, in prolactinomas harbored by lacDrd2KO female mice, not only *Notch1* and *Notch3* mRNA levels but also NOTCH2-3 membrane and NOTCH1 active domains were highly expressed in knockout mice compared to their control counterparts, the *Drd2^loxP/loxP^* mice. Even so similar subcellular location was found as specific NOTCH1-3 staining was found mainly in the cytoplasm and in some cell membranes both in lacDrd2KO and *Drd2^loxP/loxP^* mice, and neither pituitary *Jagged1* nor *Dll1* ligands showed any differences between genotypes. Similarly, Lu and co-workers found no differences when evaluating *JAGGED1* mRNA levels comparing human prolactinomas and control pituitaries [46]. Instead, it has been described that other ligands, such as *Dll1* and *Dll4*, were underexpressed in non functioning and prolactin secreting adenomas [43, 44], and overexpressed in corticotropinomas [48] respectively, pointing to a specific Notch system profile for different pituitary adenomas histotypes.

A significant reduction in *Hes1* mRNA levels was found in the anterior pituitaries of the lacDrd2KO mice bearing prolactinomas compared to *Drd2^loxP/loxP^* mice. In concordance, decreased *HES1* mRNA levels have been described both in prolactinomas and non functioning adenomas compared to normal human pituitaries [44].

Therefore, our results suggest that the generation of prolactinomas evoked by disruption of lactotrope dopamine D2R receptors is associated with high expression of some Notch components, particularly Notch receptors; these results should be highlighted in the search of new targets for dopamine agonist resistant prolactinomas.

Published data of Notch participation on pituitary adenoma generation and progression are scarce. Based on our present results of differential expression in pituitary adenomas, cell lines and normal pituitaries it becomes evident that Notch system involvement in pituitary tumorigenesis is dependent on each tumor type, and specially activated in corticotrope tumors. Further studies are needed to precisely unravel the most important components that participate in each adenoma histotype, and the processes affected by Notch pathways during pituitary tumor development and maintenance.

## MATERIALS AND METHODS

### Patient samples

Human pituitary adenoma samples were obtained from patients derived to pituitary neurosurgery at La Pequeña Familia Clinic in Junín and Santa Isabel Clinic, in Buenos Aires, Argentina. Adenomas were previously classified according to hormone production in somatotropinomas, prolactinomas, corticotropinomas and non functioning adenomas, when no hormone secretion was detected. Table 1 and Table 2 describe clinical features, tumor samples and their corresponding Ki67 values.

Samples were conserved in RNAlater (Ambion Inc) for mRNA determination or immediately fixed after surgery in 4 % neutral buffered formalin, dehydrated in graded ethanol and embedded in paraffin. Sections of 4 μm thickness were cut and immunohistochemistry for different antigens was performed.

The project and procedures were approved by the Ethical Committees of the La Pequeña Familia and Santa Isabel Clinics. The patient was informed of the studies to be undertaken and signed the respective consent. Patient privacy was always preserved.

### Cell lines and culture

AtT20 mouse corticotrope and GH3 rat somato-prolactinoma cell lines were cultured in adhesion in DMEM/F12 medium, supplemented by 10% fetal bovine serum, 1% glutamine and 1% penicillin/streptomycin and maintained at 37°C and 5% CO_2_. Rat prolactinoma MMQ cells were cultured in suspension in DMEM/F12 medium with 10% horse serum, 5% fetal bovine serum, 1% glutamine and 1% penicillin/streptomycin and maintained at 37°C and 5% CO_2_.

### Animals

### Mice

Mice lacking expression of D2Rs in pituitary lactotropes were generated by crossing *Drd2^loxP/loxP^* mice [59] with transgenic mice expressing *Cre* recombinase driven by the mouse prolactin promoter (Tg(Prl-cre)^1Mrub^ [60]) for two generations. Tissue specificity of *Cre* expression in (Tg(Prl-cre)^1Mrub^ transgenic mice was analyzed by real time PCR and *Cre* mRNA levels were highly expressed in the pituitary lactotropes and very low or almost absent in the hypothalamus, liver, kidney, ovary and lung [61]. Functional Cre recombinase activity is present in most prolactin producing cells of the anterior pituitary in a highly selective manner as described [60]. LacDrd2KO and their *Drd2^loxP/loxP^* control littermates were congenic to C57BL/6J (*n* = 10).

Breeding pairs of female *Drd2^loxP/loxP^* and male *Drd2^loxP/loxP^.Tg(Prl-Cre)* mice were used to generate *Drd2^loxP/loxP^* (control) and *Drd2^loxP/loxP^.Tg(Prl-Cre)* (lacDrd2KO) littermates, which were included in each experiment. Female 10 month-old mice were used in the present experiments.

Normal pituitaries from adult female BalbC mice were used as controls in the determination of mRNA expression by qRT-PCR and protein expression by Western blot in comparison with AtT20 cells.

Xenotropic tumors were generated by sc injection of 500,000 GH3 cells in Nude/Nude mice. Tumors were allowed to develop for 21 days, thereafter animals were euthanized and tumors were excised and collected in RNA later for qRT-PCR or in lysis buffer for protein extraction and Western blot experiments

### Rats

Female Sprague-Dawley rats were used. Animals were euthanized at two months of age. Pituitaries were used as controls in the determination of mRNA expression by qRT-PCR and protein expression by Western blot in comparison with rat tumor cell lines and GH3 *in vivo* tumors.

All animals (mice and rats) were housed in groups of 4 or 5 in a temperature-controlled room with light on at 0700 h and off at 1900 h, and had free access to tap water and laboratory chow. Experimental procedures were carried according to guidelines of the institutional animal care and use committee of the National University of the Northwest of Buenos Aires Province and Instituto de Biología y Medicina Experimental – CONICET.

### Reagents

Unless otherwise specified, all chemicals were purchased from Sigma (St. Louis, MO).

### RNA extraction and cDNA synthesis

Total RNA extraction from human pituitary tumors, mouse or rat pituitaries or from 6 × 10^5^ tumor cells was recovered using TRI reagent (Molecular Research Center, Inc) as previously described [62]. The RNA concentration was determined on the basis of absorbance at 260 nm, its purity was evaluated by the ratio of absorbance at 260/280 nm (∼2.0 was considered appropriate), and its integrity was evaluated by agarose gel electrophoresis. RNA was kept frozen at −80°C until analyzed. One μg of RNA was reversed transcribed in 20 μl volume in the presence of 3 mM MgCl_2_, 50 mM Tris·HCl (pH 8.3), 75 mM KCl, 1 mM deoxy-NTPs, 0,01 mM DTT, 1 pM oligo(dT)_15–18_ primer (Biodynamics, Buenos Aires, Argentina), and 10 U of MMLV reverse transcriptase (Invitrogen, CA, USA). The reverse transcriptase was omitted in negative reactions.

### Real time PCR

Quantitative PCR was performed as previously described in [63]. Sense and antisense oligonucleotide primers were designed by the use of PrimerBlast (http://www.ncbi.nlm.nih.gov/tools/primer-blast/). Oligonucleotides were synthesized by Biodynamics SRL and their sequences and annealing temperatures are described in Supplementary Table 1.

Briefly, the reactions were performed by kinetic PCR using 7.5 ul FastStart SYBR Green Master Mix (containing FastStart Taq DNA Polymerase, Reaction Buffer, Nucleotides, and SYBR Green I, Roche Laboratories), 150 ng cDNA and 0.5 μM primers in a final volume of 15 μl. After denaturation at 95°C during 15 min, the cDNA products were amplified with 40 cycles 20 s at 95°C, 60 s at 55–65,5°C depending on the primer pair, and 40 s at 72°C. The accumulating DNA products were monitored by the LineGene9600 (Bioer, Binjiang, China), and data were stored continuously during the reaction. Results were validated on the basis of the quality of dissociation curves as described in [63], and target gene expression relative to G*apdh* mRNA was calculated as previous published work [63]. When studying patient samples, qPCRs were performed sequentially, and in some samples not all Notch receptors, target or downstream genes could be measured due to limitations of cDNA availability.

### Western blot

Pituitary samples and cells lysates were prepared in a motor microtissue mixer in 80 to 300 μL of lysis buffer (50 mM HEPES [pH 7.4], 140 mM NaCl, 10% glycerol, 1 mM EDTA, 1 mM sodium orthovanadate, 10 mM sodium pyrophosphate, 100 mM sodium fluoride (NaF), and 10 μg/mL 1% Triton X-100) and 1-mM phenymethylsulfonyflouride (PMSF), and protease cocktail inhibitor (Roche Diagnostic, Mannheim, Germany) was added to the buffer just before use. The homogenate was then centrifuged at 12.000 rpm for 30 min at 4°C. An aliquot of the supernatant was taken to quantify proteins by the Qubit Quant-it protein assay kit (Invitrogen, Buenos Aires, Argentina).

Forty micrograms of proteins in 20 μl of homogenization buffer were mixed with 5 μl of 5x sample buffer (312 mM Tris.HCl, 10% SDS, 25% glycerol, 0.002% bromophenol blue and 1% Beta-mercaptoethanol, pH 6.8). Samples were heated 5 min at 95°C and separated by 10% sodium dodecyl sulphate-polyacrilamide gel electrophoresis (SDS–PAGE) and electrotransferred to nitrocellulose membranes (G&E, Little Chalfont, UK). After blocking with 3% nonfat dry milk solution in phosphate saline buffer - Tween (PBST) (10 mM sodium phosphate, 2 mM potassium phosphate [pH 7.4], 140 mM NaCl, 3 mM KCl, and 0.1% Tween 20) blots were incubated overnight at 4°C with primary antibodies. Rabbit polyclonal anti-Notch 1 [1:1000] and rabbit polyclonal anti-Notch 3 [1:1000], Santa Cruz Biotechnology Inc (Texas, USA) and rabbit polyclonal anti-Notch 2 [1:1000], Merck Millipore (Darmstadt, Germany) were used.

Membranes were washed with PBST and incubated with the corresponding horse raddish peroxidase (HRP)-conjugated secondary antibody (Santa Cruz Biotechnology Inc), and protein bands were visualized on ImageQuant LAS 4000 mini (G&E). The monoclonal anti-β-actin (Santa Cruz Biotechnology Inc) antibody [1:1500] was used to validate equal amount of protein loaded and transferred.

For repeated immunoblotting, membranes were incubated in stripping buffer (62.5 mM Tris, 2% sodium dodecyl sulfate, and 100 mM mercaptoethanol, pH 6.7) along 40 min at 55°C and reprobed. Band intensities were quantified using ImageJ software (National Institutes of Health, Bethesda, MD).

NOTCH1-3 expression levels were evaluated by the semi-quantification of two bands, the active intracellular domain (NICD) of 80 KDa and the cleaved membrane domain of the receptor which is still at the cellular membrane (NTMIC) of 110 KDa.

### Immunohistochemistry

Immunohistochemistry of paraffin embedded pituitaries from 10- month-old *LacDrd2KO* and *Drd2^loxP/loxP^* female mice or human pituitary tumor samples, was performed as previously described [64].

Briefly, antigen retrieval procedure was performed in citrate buffer (10 mM, pH = 6) and the microwave technique. Tissues were exposed to the primary antibody overnight at 4°C. Replacement of the primary antibody with phosphate buffer was employed as a negative control. Subsequently, slides were washed to eliminate antibody excess and incubated with the appropriate secondary antibody and then with streptavidin/biotin peroxidase complex. Diaminobenzidine was used as chromogen.

For mouse tissue the following antibodies were used: Rabbit polyclonal antibody against NOTCH1 and NOTCH2 (dilution 1:700, Merck Millipore), NOTCH3 (dilution 1:200, Santa Cruz Biotechnology Inc), HES1 (dilution 1:400, Merck Millipore). For human tissue the same rabbit polyclonal antibody against NOTCH3 (dilution 1:200, Santa Cruz Biotechnology Inc) was used. Biotin-conjugated secondary goat anti-rabbit IgG antibody (dilution 1:100; Santa Cruz Biotechnology Inc) was used.

Samples were counterstained with hematoxylin and mounted with permanent mounting medium. Morphometric analysis was performed using a Carl Zeiss transmitted light microscope at 400 and 1000 total magnification.

### Statistical analyses

Normal data distribution and variance homogeneity was tested in all cases. Correlations between each *NOTCH* receptor and *JAGGED1* or *HES1* expression in human pituitaries adenomas were tested by Spearman non parametric test. Gene expression of Notch receptors, ligands and targets in AtT20 vs control pituitaries and lacDrd2KO vs *Drd2^loxP/loxP^* mice were analyzed by Student's *t*-test. Analysis of variance (ANOVA) followed by LSD Fisher test was used to analyze Notch receptor, ligand and target gene expression in rat tumoral models vs control pituitary.

Results are expressed as means ± SEM. *p* < 0.05 was considered significant.

## SUPPLEMENTARY TABLE


