# Supplementary material for: Notch system is differentially expressed and activated in pituitary adenomas of distinct histotype, tumor cell lines and normal pituitaries
Source: Oncotarget. 2017 Jul 6;8(34):57072–88. doi: 10.18632/oncotarget.19046 (PMC5593626; doi:10.18632/oncotarget.19046)
Supplement: Supplementary file 1 [file oncotarget-08-57072-s001.pdf]

# Notch system is differentially expressed and activated in pituitary adenomas of distinct histotype, tumor cell lines and normal pituitaries

## SUPPLEMENTARY MATERIALS

### Supplementary Table 1: Sequences and annealing temperatures of oligonucleotides used

| Primers | Sequences | Annealing Temperature |
|---|---|---|
| *Notch1* mouse | Fo = CAAGAGGCTTGAGATGCTCC<br>Re = GATTGGAGTCCTGGCATCGT | 56,4°C |
| *Notch2* mouse | Fo = AGTACATTTGCACCTGCCCA<br>Re = ACTGTTGGCCATAGCACACT | 57°C |
| *Notch3* mouse | Fo = GCATACTCGGGACTGCCTAC<br>Re = CAGAATGGCGGGACACAGT | 59°C |
| *Notch4* mouse | Fo = GGAAGTCCTGGCTGTGTCC<br>Re = CTACAGGTCAACCCCATGTAGC | 57,7°C |
| *Hes1* mouse | Fo = GTCAACACGACACCGGACA<br>Re = GGAATGCCGGGAGCTATCT | 57,25°C |
| *Hes5* mouse | Fo = AGGAGAAAAACCGACTGCGGA<br>Re = GGAGTAGCCCTCGCTGTAGT | 58,6°C |
| *Hey2* mouse | Fo = TGAGGTCCAATTCACCGACA<br>Re = GCATCAAAGTAGCCTTTACCC | 55°C |
| *Delta like1* mouse | Fo = GAGAAGATCGCCCCAACACT<br>Re = CCATCTTACACCTCAGTCGCT | 56,8°C |
| *Jagged1* mouse | Fo = AGACAACTGGTATCGGTGCG<br>Re = CCTGAAACTTCATGGCACTTGG | 56,9°C |
| *Gapdh* mouse | Fo = GGGGCTGCCCAGAACATCAT<br>Re = GCCTGCTTCACCACCTTCTTG | 65,5°C |
| *NOTCH1* human | Fo = GACAGCCTCAACGGGTACAA<br>Re = CACACGTAGCCACTGGTCAT | 57,2°C |
| *NOTCH2* human | Fo = CGAGTGTGTCCCAGGCTATC<br>Re = CTTCACAGAGTAGGCCCCGA | 57,95°C |
| *NOTCH3* human | Fo = CAAGCGGCTAAAGGTAGAGGAG<br>Re = CCAGCATTAGCGGGGTGAAG | 57,8°C |
| *JAGGED1* human | Fo = TGTGAAATTGCTGAGCACGC<br>Re = CCCAGGGAGGTCTCCTTACA | 57,35°C |
| *HES1* human | Fo = TGTCAACACGACACCGGATA<br>Re = ATGCCGCGAGCTATCTTTCTT | 56,5°C |
| *GAPDH* human | Fo = TGATGACATCAAGAAGGTGGTGAA<br>Re = TCCTTGGAGGCCATGTAGGCCAT | 62°C |
| *Notch1* rat | Fo = GAACAATGTGGACGCTGCTG<br>Re = CCAGCAACACTTTGGCAGTC | 58°C |
| *Notch2* rat | Fo = TTTGCAGTGTCGAGGTGGTC<br>Re = AGCGGTTCTTCTCACAAGGG | 58°C |
| *Notch3* rat | Fo = AATGTGGAGGCTACCTTGGC<br>Re = TCTCCCGGTTGGCAAAATGA | 58°C |
| *Notch4* rat | Fo = GCTCTGTCCGCCTTCTTTCT<br>Re = TTGGCTGAGCAGAAGTCTCG | 58°C |
| *Delta like1* rat | Fo = GGGCTTCTCTGGCTTCAACT<br>Re = GACATCGGCACAGGTAGGAG | 58°C |
| *Jagged1* rat | Fo = TATGCCTGCGACCAGAATGG<br>Re = AGTCACCTGGGAGTTTGCAG | 58°C |
| *Hes1* rat | Fo = ATGACAGTGAAGCACCTCCG<br>Re = GTCACCTCGTTCATGCACTC | 56°C |
| *Hey1* rat | Fo = AGCGCAGACGAGAATGGAAA<br>Re = CGCTTCTCGATGATGCCTCT | 58°C |
| *Hey2* rat | Fo = GGGAAAGGCTACTTTGACGC<br>Re = TCGCCACTTCTGTCAAGCAT | 56°C |
| *Gapdh* rat | Fo = GAAGGTCGGTGTGAACG<br>Re = TGGGTAGAGTCATACTGGAA | 58°C |
